# Integrating Nutrition into Outpatient Oncology Care—A Pilot Trial of the NutriCare Program

**DOI:** 10.3390/nu12113590

**Published:** 2020-11-23

**Authors:** Laura Keaver, Ioanna Yiannakou, Fang Fang Zhang

**Affiliations:** 1 Department of Health and Nutritional Science, Institute of Technology Sligo, FP1 YW50 Sligo, Ireland; 2 Friedman School of Nutrition Science and Policy, Tufts University, Boston, MA 02111, USA; Fang_Fang.Zhang@tufts.edu; 3 Department of Medicine, Boston University, Boston, MA 02215, USA; ioannay@bu.edu

**Keywords:** diet quality, cancer survivor, nutrition intervention, oncology care, quality of life

## Abstract

Nutrition is an essential part of oncology care; however, nutrition advice and guidance are not always provided. This six-week pilot pretest-posttest intervention was designed to test the feasibility and effectiveness of integrating a nutrition education program (NutriCare) into outpatient oncology care. Twenty breast cancer survivors were recruited through Tufts Medical Centre. Nutrition impact symptoms and demographics were collected at baseline, dietary quality and quality of life measures were collected pre and post-intervention and an evaluation form was completed post-intervention. Forty-four percent of eligible participants were recruited, and 90% of those completed the study. The NutriCare program was well received with participants reporting that goals were feasible (94.4%), the program had a positive impact on their diet (77.8%), and over 80% would recommend the program. There was an interest in continuing with the program (89%) and in receiving additional guidance from the healthcare team (83%). There was a significant improvement (*p* = 0.04) in physical function over the six weeks; however, no additional significant differences in quality of life or dietary quality were seen. In conclusion, cancer survivors were positive about the NutriCare program and its integration into practice.

## 1. Introduction

Nutrition is extremely important in the management of cancer. It is well recognized that some treatments could have detrimental effects on patients’ dietary intake [1,2], which can negatively impact the patient’s nutritional status and outcomes. It is also known that maintaining muscle mass is of utmost importance as reduced muscle mass as a result of cachexia [3] and/or sarcopenia is associated with fatigue, impaired physical function, reduced tolerance to treatments, impaired quality of life and reduced survival [4]. Therefore, it is important to deliver this information to all rather than waiting until a patient requests it or they have experienced substantial weight loss. However, in some cases, muscle wasting is not evident, for example, in those who have obesity [4]. Recent work has shown that cancer survivors who received nutritional information more often changed their dietary behavior, regardless of whether they had nutritional information needs [5]. Survivors, when compared to the general population, have a diet of poorer quality [6] and they can experience weight gain from early in treatment right into survivorship [7]. As cancer survivors are already at an increased risk of additional cardiovascular disease risk factors such as hypertension and type 2 diabetes [8], it is important that this is addressed early on to ensure the best outcomes in terms of recurrence and development of additional conditions.

Continued active clinical support and education for cancer survivors should be considered an essential element in the cancer journey to address patient well-being [9]. The NutriCare program was developed to address this gap by proving easy to use evidence-based nutrition information and a step-by-step process for clinicians and healthcare professionals to follow in order to ensure that oncology patients and survivors receive nutrition advice and guidance as part of oncology care. The aim of this study was to determine the feasibility of the NutriCare program as well as any changes in quality of life and dietary quality that occurred over the course of the six-week pilot intervention.

## 2. Materials and Methods

### 2.1. Study Design and Population

NutriCare is a program designed to integrate nutrition into oncology care. The components of this intervention have been published elsewhere [10] and have been briefly outlined below. This pilot consisted of a six-week oncology clinic study to determine the feasibility of integrating this model into outpatient oncology care. The study design was a pretest-posttest intervention. Study recruitment took place at Tufts Medical Centre through the oncology team at the Breast Health Clinic between July and August 2018, and 20 women were enrolled in the study. The inclusion criteria were (1) 21 years or older, (2) not undergoing active treatment, (3) not palliative (4) not currently enrolled in a weight management program or receiving nutritional counseling and (5) could read and speak English. Any advanced nutrition-related issues or a requirement for supplements or enteral feeding were to be referred to a dietitian, as outlined in the Health Care Professional (HCP) toolkit.

### 2.2. Intervention

**Figure 1:**
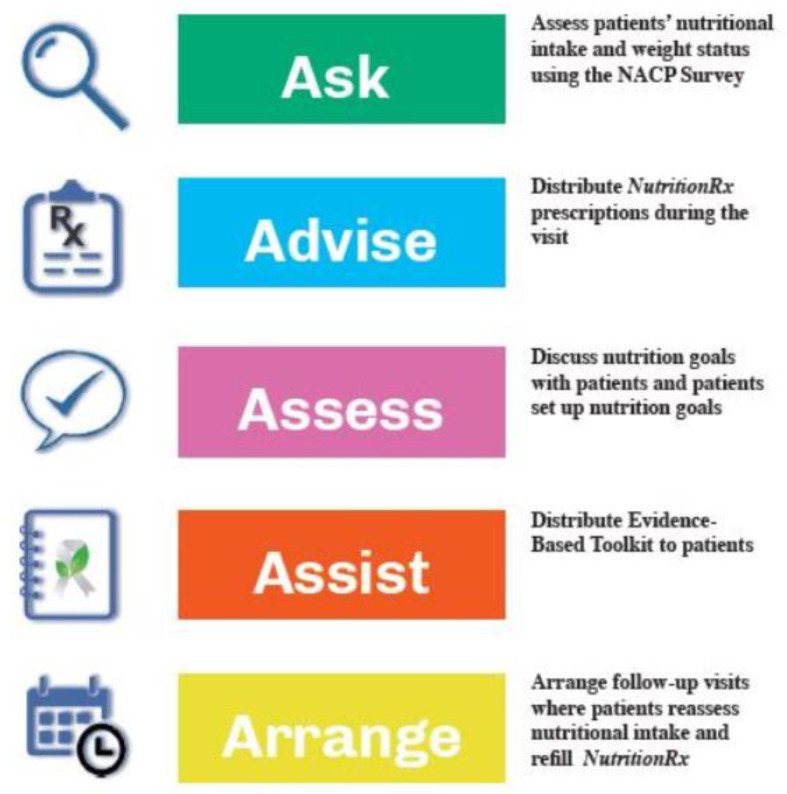
NutriCare Program Flowchart.

**Figure 2:**
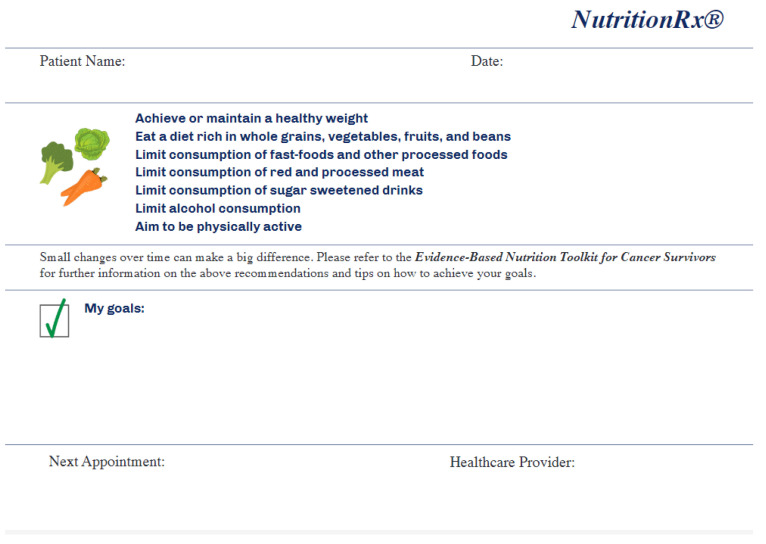
Nutrition Prescription Pad.

As mentioned previously, the NutriCare model has previously been reported, but in short, it utilizes the 5-A model (Ask, Advise, Assess, Assist, Arrange) to integrate nutrition care into the oncology setting. This model has previously been utilized in smoking cessation [11], obesity counseling [12], and the development of nutrition interventions for adolescent athletes [13]. We refined this model based on feedback from focus groups [10], and an overview of the process used in this pilot can be seen in Figure 1. It was initially designed to be fully delivered by the oncology team, however, based on feedback, it was refined to be introduced by the oncology team and was then delivered by a registered dietitian (LK). A Nutrition Assessment for Cancer Patients (NACP) questionnaire was completed by all individuals at baseline, and this formed the basis of the intervention. The symptoms list was chosen from nutrition impact symptoms commonly reported by cancer patients [14,15]. Questions on food groups were adapted from the validated Rapid Eating Assessments for Patients (REAP) [16]. Each individual then set their own Specific, Measurable, Achievable, Realistic and Timely (SMART) goals with guidance from the dietitian, and these were noted on a Nutrition prescription pad (Figure 2). A patient toolkit was provided to all participants to help them to implement these goals. This toolkit consisted of the following nutritional and educational sections: why is nutrition important for the cancer survivor; how does cancer treatment impact eating patterns; strategies for managing eating problems during cancer treatment; maintaining a healthy weight during and after cancer treatment; healthy eating and active living after cancer treatment: nutrition recommendations for cancer survivors; frequently asked questions around healthy eating (this section was divided into 15 sections comprising: plant-based diet, fruits and vegetables, wholegrain and fiber, dairy, protein, animal-based protein, plant-based protein, fats, sugar and sugary drinks, sodium, drinks, nutrition labeling, supplements, fad diets, portion control); food safety; how to talk to your doctor about diet; how to evaluate nutrition information for cancer survivors and links to additional evidence-based resources. The oncology team followed up with a phone call within seven days of the baseline visit to reinforce the goals and to continue to champion and support the intervention. Participants were provided with a parking voucher and $25 gift card to compensate them for their time.

### 2.3. Intervention Measures

An information sheet was provided to all participants by the oncology team in Tufts Medical Centre prior to consent. All participants gave informed written consent before commencing any aspect of the study. The baseline visit coincided with a clinical visit to the Breast Clinic to make it more convenient for the participants. After consent, participants provided information on socioeconomic demographics as well as completing two quality of life questionnaires – the Patient-Reported Outcome Measurement Information System (PROMIS) -57 Profile v2.1 [17] and PROMIS Scale v1.2-Global Health [18]. They were also provided with unique login details to complete the National Institutes of Health Diet History Questionnaire (DHQ) III online [19]. This questionnaire asks participants to report their average consumption of a variety of foods in the last month. A unique identification number was given to all participants to maintain confidentiality. Height was self-reported while weight was measured as a standard part of care during the medical appointment, which coincided with the study’s baseline visit and so this measurement was used.

Follow-up was six weeks after the in-person visit and consisted of a brief phone call and the mailing of the two quality of life questionnaires to be recompleted and new login details being provided to complete the DHQ III online again. The program’s feasibility was assessed in a number of ways, including using participants’ satisfaction ratings on an evaluation form provided at the six weeks follow-up, using the participants’ ratings for understanding and ease of use of the provided resources, and the study’s retention rates.

### 2.4. Data Analysis

The PROMIS -57 Profile v2.1 [17] and PROMIS Scale v1.2-Global Health [18] were both scored using the reference scoring manuals [20,21] to convert raw scores to T-scores ±SE.

For the components of the PROMIS -57 Profile v2.1 questionnaire, for the United States general population, a score of 50 is average with a standard deviation of 10. For concepts within this questionnaire that are positively worded, therefore, such as mobility, a T-score of 60 represents one standard deviation above average and a T-score of 40 represents one standard deviation below average. Conversely, with negatively worded concepts such as anxiety, a T-score of 60 represents one standard deviation below average, while a T-score of 40 represents one standard deviation above average [22].

The PROMIS Scale v1.2-Global Health gives values for both Global Mental and Global Physical Health. When reporting on Global Mental Health the cut-offs for poor, fair, good, very good and excellent are 29, 40, 48 and 56, respectively [23]. For Global Physical Health the cut-offs for poor, fair, good, very good and excellent are 35, 42, 50 and 58, respectively [23].

Dietary quality was assessed using the Healthy Eating Index (HEI)-2015 and its component score calculated by the Diet*Calc software developed by the National Cancer Institute [19], based on the dietary data collected in the DHQ III. The overall dietary quality uses a 100-point scale, with a higher score indicating a better dietary quality [24]. Thirteen components sum to make this total score of 100.

### 2.5. Statistical Analysis

Data were analyzed using SPSS version 24. Descriptive statistics were used to describe the demographic and health information and are presented as means, SDs, ranges, frequencies and percentages. The study was not powered to detect statistically significant differences, but pre-post intervention differences in dietary intake and quality of life measures were assessed using t-tests. Significance was set at *p* < 0.05.

### 2.6. Ethical Approval

This project was approved by the Institutional Review Board of Tufts University (Institutional Review Board Protocol No. 12954). The researchers obtained written informed consent from all study participants prior to enrolment in the study. All data was stored in password-protected computers and locked filing cabinets in Tufts University and only the first author and principal investigator had access to this data.

## 3. Results

### 3.1. Study Sample

Table 1 describes the characteristics of the individuals who took part in this pilot trial. Age ranged from 42 to 80 years of age with a mean (±SD) of 59.5 years (±9.9). 45% were within five years of diagnosis. The mean (±SD) of BMI was 30.2 kg/m^2^ (±6.4). 30% of the cohort reported weight gain since diagnosis, and 15% reported weight loss in this timeframe. 80% previously underwent surgery, 75% underwent chemotherapy, and 75% underwent radiation therapy. 75% were receiving hormonal therapy at the time of the pilot. Additional characteristics can be found in Supplementary Table S1.

### 3.2. Intervention

#### 3.2.1. NACP Questionnaire

**Figure 3:**
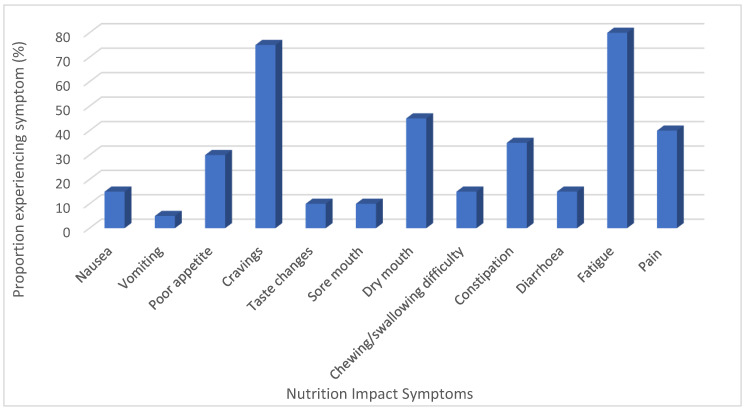
% of participants who reported experiencing nutrition impact symptoms.

The results of the NACP questionnaires are outlined in Figure 3 and Supplementary Table S2. Fatigue (80%) and cravings (75%) were the most widely reported symptoms still experienced (Figure 3). The majority of participants (80%) were very willing to make changes to their current habits to improve their health (Supplementary Table S2).

#### 3.2.2. Goals Chosen

The main goals chosen based on the results of the NACP (Figure 3 and Supplementary Table S2) and in consultation with the participants focused on tips to help manage fatigue (*n* = 8), increase vegetables by one portion on most days (*n* = 5), increase dairy by one portion on most days (*n* = 7), have breakfast (*n* = 6), include an extra portion of oily fish per week (*n* = 4), increase fruit by one portion on most days (*n* = 4) and increase fiber by introducing more wholegrains (*n* = 4).

### 3.3. Outcomes

Physical health scores ranged from 39.8 (fair) to 57.7 (very good) at baseline and from 39.8 (fair) to 67.7 (excellent) at follow-up. Mental health scores ranged from 31.3 (fair) to 67.7 (excellent) at baseline and from 33.8 (fair) to 67.7 (excellent) at follow-up (Table 2).

There was a statistically significant improvement in physical function over the course of the six-week intervention (*p* = 0.04). There were no additional statistically significant changes in quality of life or in overall dietary quality as measured by the HEI-2015 and its component scores from pre-test to post-test (Table 2).

### 3.4. Process Feasibility

#### 3.4.1. Recruitment and Retention

A total of 46 participants were deemed eligible for inclusion by the oncology medical team. We were unable to make contact with 14 of these, 12 declined to participate citing time restraints and 20 agreed to participate (44% of those eligible) and were enrolled in the study. In all, 18 of these 20 participants (90%) completed post-testing.

#### 3.4.2. Satisfaction and Acceptability

Participants were very positive about the NutriCare program (Table 3). They reported that goals were feasible (94.4%), that the program had a positive impact on their diet (77.8%), and over 80% reported that locating information was easy, the tips were practical and useful and that they would recommend the program to anyone with cancer. There was a strong commitment to continuing to use the toolkit provided (89%) and also a strong desire for the oncology team to continue to include nutrition as a topic during future consultations (83%).

### 3.5. Management Feasibility

Recruitment of participants occurred primarily by phone which proved difficult as not everyone could be reached or responded to phone messages. Focus groups to refine this program were carried out prior to implementation [10] and these highlighted the role of the doctor should be to champion this program. As such, the doctor introduced the program to all participants initially. Additionally, the oncology team followed up with phone calls to reiterate the importance of nutrition. These phone calls took approximately 10 min and feedback from the medical team reported that it was not feasible (logistic-wise) to reach patients by phone to discuss nutrition. The team only managed to contact over half (*n* = 10, 55.6%) of the enrolled participants by phone to follow-up (Table 3).

## 4. Discussion

The NutriCare program was well-received by breast cancer survivors in this study. There was an interest in continuing with the program and in receiving additional guidance from HCPs. There was a significant improvement (*p* = 0.04) in physical function over the six weeks; however no additional significant differences in quality of life or dietary quality were seen.

Enrollment in this study from a breast cancer clinic showed that about 44% of those eligible were contactable and willing to take part in this pilot intervention. Recruitment/response rates in previous studies with cancer survivors have not always been reported; however, where available they range from 3–81% [25,26,27,28]. The majority of interventions tend to focus disproportionately on breast cancer survivors [29,30]. While 90% of those enrolled completed this intervention, it was short in duration (six weeks) and recruited a highly motivated cohort. However, several previous studies have reported similar rates [25,26] for studies of longer duration (up to 12 months). Rates of attrition range from 0–50% in intervention studies with cancer survivors [29].

There was an interest in continuing to receive nutrition advice in the oncology setting and in continuing with the program. Cancer survivors have previously demonstrated an interest in modifying their dietary intake and physical activity levels in the hopes of preventing recurrence [31]. The involvement of the healthcare team, and in particular the oncologist in interventions, has been shown to influence the perceived behavioral control of the patient and to lead to improvements in the desired behavior [31]. Given the time and confidence in one’s nutrition knowledge that inclusion of additional components into standard appointments can take [32,33] and the difficulty the medical team had in this study when trying to follow up with a phone call, it is worth considering how else this intervention could be delivered and supported by the oncology team. Interventions delivered online, particularly when supported with a variety of behavior change techniques have shown promise [34,35]. This would also help to address an additional barrier that has frequently been reported by cancer survivors, that of distance/traveling [29,36].

This pilot was not designed to test effectiveness but to demonstrate feasibility and acceptability and inform a future larger-scale trial. Still, our pilot data suggest an improvement in physical function after six weeks. A decline in physical functioning has been associated with loss of mobility, loss of independence, an increased risk of adverse outcomes, and mortality [37]. As cancer survivors are at an increased risk of losing physical functioning as they age [38,39,40], interventions that can improve this are needed. Typically, physical activity is recommended to prevent loss of physical function; however, given that only 30% of cancer survivors achieve recommended physical activity levels [41], it is important to consider the role of other lifestyle behaviors. A higher dietary quality (as seen in this group) has been strongly associated with better physical functioning and decreased odds of functional impairment [42].

Overall, the dietary quality of this group was good. Our findings are consistent with previous research that has shown dietary quality to be higher in well-educated cohorts of a high socioeconomic status compared to those who are less well educated and/or from a lower socioeconomic status [43,44,45]. However, it contrasts with a study of 1533 cancer survivors from the United States where overall dietary quality, as measured by HEI was shown to be 47.2 (±0.5) [6]. Assessing dietary quality proved challenging as using the Dietary Health Questionnaire, which is quite long had an impact on the number who were willing to complete it. Only 11 of the 20 completed this measure and so results should be interpreted with caution. There is also a chance that those who had a higher quality diet and overall better lifestyle behaviors were more likely to complete this measure [46].

Despite the fact that 55% of the cohort had been diagnosed greater than five years ago, fatigue and cravings were two nutrition impact symptoms that still affected the majority (80% and 75%, respectively). The impact that high levels of fatigue, as demonstrated in this group, could have on the ability to shop for and prepare food and therefore, overall dietary intake should not be ignored. A large meta-analysis of 12, 327 breast cancer survivors who had completed treatment reported that one in four suffered from severe fatigue post-treatment, with the prevalence reducing substantially in the first 6 months after treatment [47]. This is similar to previous work, which indicated that one-year post-treatment fatigue levels had returned to pre-treatment levels [48,49]. In contrast to this, prevalence rates of 38–66% have been reported in disease-free breast cancer survivors [50,51,52] with fatigue being reported in up to one-third of breast cancer survivors five to ten years after completion of treatment [53,54,55]. As the number of individuals surviving cancer increases, it is important to further improve our knowledge of the persistence of symptoms post-treatment. This information will help inform the development of nutrition guidelines and advice specific for cancer survivors (of specific cancer types), whose needs are quite different from those in the initial stages of treatment. One interesting area of exploration is the finding that diets high in fiber have been linked to reduced levels of fatigue in breast cancer survivors [56].

There were a number of limitations in the study. First, the survivors who enrolled in the study were highly educated, white, breast cancer survivors of high socioeconomic status and so the results of this pilot will not be applicable to broader cancer groups, cancer types or ethnic groups. This bias has been demonstrated regularly in previous work also [29,30]. In addition, the intervention took place over a short duration of time (six weeks). While this was designed to allow for the feasibility and acceptability of such a program to be determined, it meant that the impact of the program on dietary quality and quality of life, which are likely to take longer than six weeks, could not fully be determined. In addition, the use of a comprehensive online dietary food frequency questionnaire resulted in a lower number of responses being returned (*n* = 11) at the six-week follow-up. We also did not assess specific laboratory values, which could have served as risk surrogates indicating short-term changes and could also have acted as motivational tools.

## 5. Conclusions

In conclusion, cancer survivors were positive about the NutriCare program and its integration into practice. There was an interest in continuation with the program and additional guidance from HCPs. There is warrant in investigating the delivery of this program through different mediums, e.g., online, to ensure that patients and survivors who are attending centers that do not have the resources to run such a program will still be able to take part and get access to evidence-based nutrition guidance.

## Figures and Tables

**Table 1:** Characteristics of the participants (*n* = 20) recruited into the NutriCare pilot trial.

	*n* (%) or Mean (SD)
Age, years, mean (SD)	59.5 (9.9)
BMI, mean (SD)	30.2 (6.4)
BMI classification, *n* (%)	
Underweight (<18.5 kg/m^2^)	0 (0)
Healthy weight (18.5–24.99 kg/m^2^)	6 (32)
Overweight (25–29.99 kg/m^2^)	3 (15)
Obese (≥30 kg/m^2^)	10 (53)
Weight gain, *since diagnosis*, *n* (%)	
Yes	6 (30)
No	14 (70)
Weight loss, *since diagnosis*, *n* (%)	
Yes	3 (15)
No	17 (85)
Diagnosis, *years since*, *n* (%)	
<5 years	9 (45)
5–10 years	7 (35)
10–15 years	1 (5)
15+ years	3 (15)
Breast cancer stage, *n* (%)	
1A	5 (25)
1B	0 (0)
2A	7 (35)
2B	3 (15)
3A	2 (10)
3B	1 (5)
3C	2 (10)
Hormonal Receptor Status, *n* (%)	
ER + PR + HER2-	10 (50)
ER-PR-HER2-	1 (5)
ER + PR + HER2 1 +	3 (15)
ER + PR + HER2 2 +	1 (5)
ER + PR + HER2 3 +	2 (10)
ER + PR + HER-	1 (5)
ER-PR-HER-	1 (5)
Previous Surgery, *n* (%)	
Yes	16 (80)
No	4 (20)
Previous Radiation, *n* (%)	
Yes	15 (75)
No	5 (25)
Years since radiation completion, mean(SD)	4.3 (4.1)
Previous Chemotherapy, *n* (%)	
Yes	15 (75)
No	5(25)
Years since chemotherapy completion, mean(SD)	6.5 (6.4)
Hormonal Therapy, *n* (%)	
Current	15 (75)
Previous	4 (20)
Never	1 (5)
Race/ethnicity, *n* (%)	
White Caucasian	18 (90)
Black/African American	1 (5)
Other	1 (5)
Education, *n* (%)	
High school	4 (20)
Associates degree	3 (15)
Some college	3 (15)
College	8 (40)
Graduate	2 (10)

**Table 2:** Changes in quality of life and dietary quality after 6 weeks intervention.

	Baseline/Pre-Test Mean (SE)	Six Weeks/Post-Test Mean (SE)	Significance/*p*-Value
**Quality of Life** (PROMIS Scale v1.2-Global Health) ^a^			
Physical	49.5 (6.0)	50.2 (6.7)	0.7
Mental	51.5 (9.4)	51.0 (7.4)	0.8
**Quality of Life** (PROMIS -57 Profile v2.1) ^b,c^			
Physical Function	48.6 (7.4)	51.8 (7.9)	0.04
Anxiety	48.3 (9.3)	46.3 (9.4)	0.5
Depression	43.5 (8.9)	45.3 (8.5)	0.3
Fatigue	48.1 (8.4)	48.2 (7.7)	0.9
Sleep disturbance	49.8 (7.8)	49.6 (6.3)	0.9
Ability to participate	55.5 (7.4)	55.9 (8.1)	0.8
Pain interference	47.3 (7.8)	49.6 (7.5)	0.1
Pain intensity	2.3 (2.5)	2.2 (2.1)	0.8
	**Baseline/pre-test** **Mean (SE)**	**Six weeks/post-test** **Mean (SE)**	
**Dietary quality (HEI-2015 component scores)** ^d^			
Total HEI-2015 score	74.3 (13.3)	74.3 (8.6)	1.0
Vegetables	4.5 (0.7)	4.4 (0.4)	0.9
Greens and beans	4.5 (1.3)	4.7 (0.8)	0.3
Total fruits	4.4 (1.2)	4.2 (1.4)	0.6
Whole fruits	4.5 (1.0)	4.7 (1.0)	0.6
Wholegrains	4.7 (2.6)	3.6 (2.0)	0.2
Dairy	6.0 (1.9)	6.7 (2.3)	0.3
Total protein foods	4.8 (0.6)	5.0 (1.4)	0.3
Seafood and plant proteins	4.7 (0.8)	4.8 (0.6)	0.3
Fatty acids	6.7 (2.8)	6.6 (2.8)	0.8
Sodium	4.7 (3.1)	4.9 (3.2)	0.9
Refined grains	9.6 (0.9)	9.5 (1.1)	0.7
Saturated fats	6.9 (3.7)	7.7 (2.5)	0.4
Added sugars	8.3 (2.0)	7.5 (1.7)	0.3
% calories from added sugars	9.2 (4.7)	10.8 (4.4)	0.4
% of calories from saturated fats	10.4 (3.2)	9.5 (2.4)	0.3

^a^ Of participants enrolled in this trial (*n* = 20), these variables were available for 19 participants at pre-test, 18 at post-test and 17 for both time-points. ^b^ Of participants enrolled in this trial (*n* = 20), these variables were available for 20 participants at pre-test, 18 at post-test and 18 for both time-points. ^c^ The range of scores for physical function were 36.7 to 60.1 at baseline and 38.1 to 60.1 at follow-up, for anxiety 37.1 to 66.6 at baseline and 37.1 to 67.7 at follow-up, for depression 38.2 to 64.9 at baseline and 38.2 to 64.9 at follow-up, for fatigue 33.1 to 61.3 at baseline and 33.1 to 62.3 at follow-up, for sleep disturbance 30.5 to 59.1 at baseline and 35.3 to 59.1 at follow-up, ability to participate 41.1 to 65.4 at baseline and 44 to 65.4 at follow-up and pain interference 40.7 to 60.8 at baseline and 40.7 to 65.5 at follow-up. ^d^ Of participants enrolled in this trial (*n* = 20), these variables were available for 17 participants at pre-test, 12 at post-test and 11 for both time-points.

**Table 3:** Participant Evaluation of the Six-Week Pilot of the NutriCare Program (*n* = 18)**.**

**Clinical Visit** *Please Answer the Following Questions using the Scale Below*			
	**Strongly Agree/Agree** ***n* (%)**	**Neutral** ***n* (%)**	**Disagree/Strongly Disagree** ***n* (%)**
I think that nutrition is an important component of my care	17 (94.4)	1 (5.6)	0 (0)
The nutrition prescription was specific to me	11 (61.1)	7 (38.9)	0 (0)
The goals set were feasible to achieve	17 (94.4)	1 (5.6)	0 (0)
Overall, this program impacted my diet positively	14 (77.8)	4 (22.2)	0 (0)
**Effectiveness and ease of use** *Please answer the following questions on the toolkit provided to you during your visit using the scale below*			
The toolkit…	**Strongly Agree/Agree** ***n* (%)**	**Neutral** ***n* (%)**	**Disagree/Strongly Disagree** ***n* (%)**
Helped me to better understand the importance of nutrition in cancer care	17 (94.4)	1 (5.6)	0 (0)
Helped me to change my diet	17 (94.4)	1 (5.6)	0 (0)
Helped me to maintain/achieve a healthy weight	12 (66.7)	6 (33.3)	0 (0)
Locating information was easy	15 (83.3)	3 (16.7)	0 (0)
The tips provided were practical and useful	15 (83.3)	3 (16.7)	0 (0)
I would recommend the toolkit to anyone with cancer	16 (88.9)	2 (11.1)	0 (0)
**Follow-up with oncology provider** *Please answer the following questions using the scale below*			
	**Yes** ***n* (%)**	**No** ***n* (%)**	**N/A** ***n* (%)**
I received a follow-up phone call from my oncology provider	10 (55.6)	2 (11.1)	6 (33.3)
This call motivated me to achieve my dietary goals	7 (38.9)	1 (5.6)	10 (55.6)
**Longer-term** *Please answer the following questions using the scale below*			
	**Strongly Agree/Agree** ***n* (%)**	**Neutral** ***n* (%)**	**Disagree/Strongly Disagree** ***n* (%)**
I would like providers to continue the nutrition conversation at future visits	15 (83.3)	3 (16.7)	0 (0)
I will continue using the toolkit	16 (88.9)	2 (11.1)	0 (0)

## Supplementary Materials

The following are available online at https://www.mdpi.com/2072-6643/12/11/3590/s1, Table S1: Additional participant characteristics; Table S2: Results of the NACP questionnaire (*n* = 20), at baseline.
