# Supplementary material for: Integrating Nutrition into Outpatient Oncology Care—A Pilot Trial of the NutriCare Program
Source: Nutrients. 2020 Nov 23;12(11):3590. doi: 10.3390/nu12113590 (PMC7700247; doi:10.3390/nu12113590)
Supplement: Supplementary file 1 [file nutrients-12-03590-s001.pdf]

Supplementary Table 1: Additional participant characteristics

	N(%) or Mean (SD)
**Diabetes Mellitus,** n(%)	
Yes	1 (5)
No	19 (95)
**Hypertension,** n(%)	
Yes	5 (25)
No	15 (75)
**Dyslipidemia,** n(%)	
Yes	3 (15)
No	17 (85)
**Income,** n(%)	
<$40,000	0 (0)
$40,000-$59,000	4 (20)
$60,000-$79,000	0 (0)
$80,000+	11 (55)
Not specified	5 (25)
**Marital Status,** n(%)	
Married	9 (45)
Divorced	2 (10)
Widowed	3 (15)
Unmarried	6 (30)
**Living Situation,** n(%)	
Lives alone	3 (15)
Lives with others	17 (85)
**Employment,** n(%)	
Full time	13 (65)
Part time	1 (5)
Self employed	1 (5)
Unemployed	1 (5)
Student	1 (5)
Retired	3 (15)
**Insurance,** n(%)	
Private	15 (75)
Public	1 (5)
Private and Public	3 (15)
Other	1 (5)

Supplementary Table 2: Results of the NACP questionnaire (n=20), at baseline

Dietary Intake				
**In an average week, how often do you**	**Usually/often n(%)**	**Sometimes n(%)**	**Rarely/never n(%)**	**Not applicable n(%)**
eat less than 2 servings of fruit per day	4 (20)	9 (45)	7 (35)	0 (0)
eat less than 2.5 servings of vegetables per day	3 (15)	8 (40)	9 (45)	0 (0)
eat less than 3 servings of wholegrains per day	7 (35)	10 (50)	3 (15)	0 (0)
eat or drink less than 3 servings of milk, yogurt or cheese per day	7 (35)	10 (50)	3 (15)	0 (0)
eat more than 6 servings of cooked red meat a week	0 (0)	5 (25)	12 (60)	3 (15)
eat any processed meats	1 (5)	5 (25)	11 (55)	3 (15)
eat less than 4 servings of nuts/seeds per week	8 (40)	3 (15)	9 (45)	0 (0)
drink 16 ounces or more of non-diet soda, fruit drink/punch or Kool-Aid per day	0 (0)	1 (5)	12 (60)	7 (35)
drink more than 1 alcohol drink per day (women) or more than 2 alcoholic drinks per day (men)	0 (0)	6 (30)	9 (45)	5 (25)
eat 4 or more meals from takeout restaurants in a week	0 (0)	4 (20)	13 (65)	3 (15)
**Motivation**				
How willing are you to make changes in your eating habits in order to be healthier?				
**Not willing at all**				**Very willing**
0 (0)	1 (5)	0 (0)	3 (15)	16 (80)
